# A Bladder Wall Angiomyolipoma as a Manifestation of Tuberous Sclerosis: First Case Report

**DOI:** 10.1155/2013/398328

**Published:** 2013-09-18

**Authors:** Mehmet Kalkan, Coşkun Şahin, Ömer Etlik, Ergun Uçmaklı

**Affiliations:** ^1^Fatih University, Sema Hospital, Department of Urology, Sahil Yolu Sok. No. 16, Dragos, Maltepe, 34844 Istanbul, Turkey; ^2^Fatih University, Sema Hospital, Department of Radiology, Istanbul, Turkey; ^3^Fatih University, Sema Hospital, Department of Pathology, Istanbul, Turkey

## Abstract

A 21-year-old female patient admitted to the emergency department complaining of left side pain. Hypovolemic shock, which was probably caused by retroperitoneal bleeding from left sided renal angiomyolipoma, was developed. Abdominal computed tomography showed multiple fat containing lesions in different, regions including right bladder wall, lower pole of left kidney, and right kidney. Some lesions compatible with tuberous sclerosis such as angiofibromas, Shagreen patches, myocardial, and brain hamartomas were also detected. Bladder wall mass showing intra- and extravesical extensions was seen at exploration. We removed the tumor completely preserving bladder trigone. Angiomyolipoma located at lower pole of left kidney was also removed. Diagnosis of bladder angiomyolipoma was confirmed by the immunohistochemical examination. Recurrent or residual mass was not detected at the three-months-follow-up. We report the first case of bladder angiomyolipoma confirmed by histopathologically as a tuberous sclerosis.

## 1. Introduction

Tuberous sclerosis (TS) is a genetic multisystem disorder characterised by prevalent hamartomas in several organs, including skin, brain, eyes, heart, kidney, lung, and liver [1]. The expression of the disease varies substantially regarding affected organs. Renal angiomyolipomas are the most common urinary system manifestation of TS.

Angiomyolipomas are rare tumors and associated with tuberous sclerosis in 20–30% of cases. The kidney is the most commonly affected organ and liver, mediastinum, colon, uterus, and lung are also be affected [2, 3].

A limited number of previous bladder angiomyolipomas have been reported in the literature. But bladder angiomyolipoma associated with tuberous sclerosis has not been seen in the literature.

 We report the first case of bladder angiomyolipoma in a 21-year-old woman with TS.

## 2. Case Report

A 21-year-old female patient was admitted to the emergency department complaining of left side pain. Hypovolemic shock, which was probably caused by retroperitoneal bleeding from left sided renal angiomyolipoma, was developed. Abdominal computed tomography showed multiple fat containing mass lesion in different regions including right bladder wall and lower pole of left kidney and right kidney. Furthermore, there was prominent hemorrhagic fluid in left retroperitoneal space. After stabilization of hemodynamic condition, selective arterial embolization was performed for the angiomyolipoma that was the cause of the bleeding. Dermatologic, cardiac, and neurologic examinations were done. Some skin lesions compatible with TS such as angiofibromas on and around the nose were observed on the face and Shagreen patches in the inguinal and gluteal regions. Cardiac lesions, which were located in myocardium suggesting hamartomas, were detected by echocardiography. In addition, subcortically located abnormal signal intensities compatible with hamartomas were detected at brain magnetic resonance imaging (MRI). But the patient had no history of cardiac complaints, epilepsy, and mental retardation symptom. The main complaint of the patient was dysuria and recurrent urinary tract infection without macroscopic hematuria.

Abdominal MRI was performed just before the surgical intervention. MRI revealed that a left sided angiomyolipoma embolised before is found to be same size, a fat containing mass lesion showing intravesical and extravesical extensions is located at right bladder wall, and a fat containing mass lesions is at the right kidney.

We made intraperitoneal exploration through lower midline incision. Bladder wall mass 10 cm in size showing intra-vesical and extravesical extensions was also seen at exploration. We made a frozen section and pathologic exam result was reported as a benign lesion. Thereafter, we established the surgical treatment strategies regarding the pathologic exam result; consequently, partial cystectomy was carried out. We removed the bladder wall tumor completely preserving bladder trigone. Angiomyolipoma, 12 cm in size, located at lower pole of left kidney, was also removed through upper midline incision in the same session. After the surgery, the patient was uneventful and no residual mass lesion on bladder wall was detected at the three-month-follow-up abdominal MRI.

## 3. Radiological Findings

**Figure 1:**
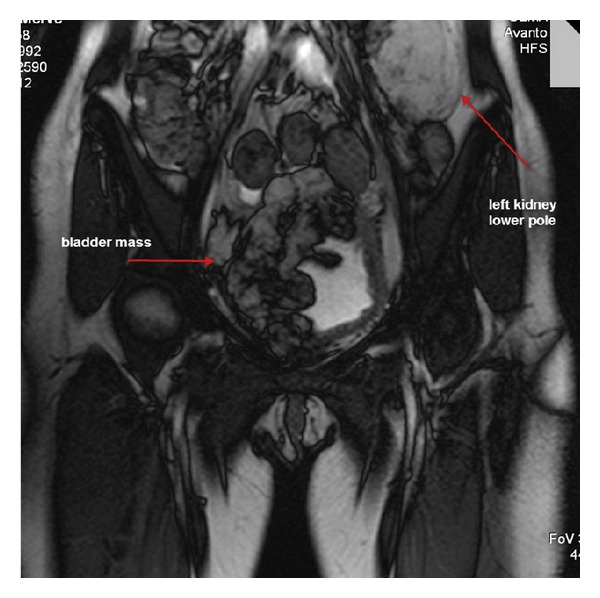
Coronal image showing a multilobulated mass lesion, arising from the right and superior walls of the bladder, largely extended in right half of the pelvis is observed.

A multilobulated mass lesion, 10 × 6 × 7 cm in size, arising from the right and superior walls of the bladder, largely extended in right half of the pelvis is observed (Figure 1). The mass lesion shows endophytic and exophytic protrusions. The mass lesion surrounds the distal part of the right ureter, iliac vein, and artery but does not lead to compression on these structures. The mass lesion shows hyperintensities corresponding to fatty tissue on T1 weighted images. These mass lesions show scattered contrast enhancement on contrast enhanced T1 weighted sequences. Contrast enhancement is related to the presence of vascular compartment in this tissue.

**Figure 2:**
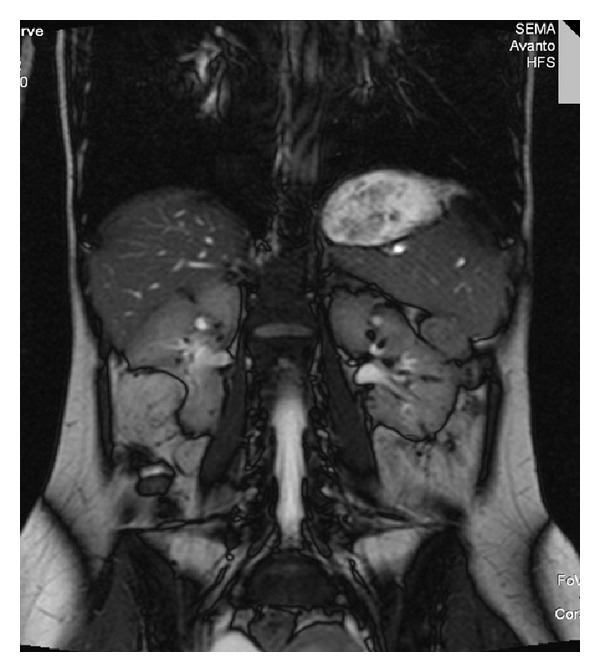
Angiomyolipoma showing hyperintensities compatible with fatty tissue on T1 weighted images is seen at lower pole of left kidney.

In Figure 2, Angiomyolipoma showing hyperintensities compatible with fatty tissue on T1 weighted images is seen at lower pole of left kidney. This lesion causes displacement of left kidney anteriorly. Note some lesions with similar radiologic characteristics at right kidney. You can see the millimetric sized hamartomas as hypointens areas on both T1 and T2 weighted images.

## 4. Pathological Findings

### 4.1. Macroscopic Assessment

#### 4.1.1. Tissue Taken from the Left Kidney Lower Pole

An 8 cm tumoral tissue, which is rich in fatty tissue, with bleeding areas, surrounded by a smooth contoured is monitored.

#### 4.1.2. Tissue Taken from the Bladder

Smooth contoured tumoral tissue containing fatty tissue, 10 × 8 × 8 cm in size, extending into the bladder lumen at the upper portion of the bladder is observed.

### 4.2. Microscopic Assessment

**Figure 3:**
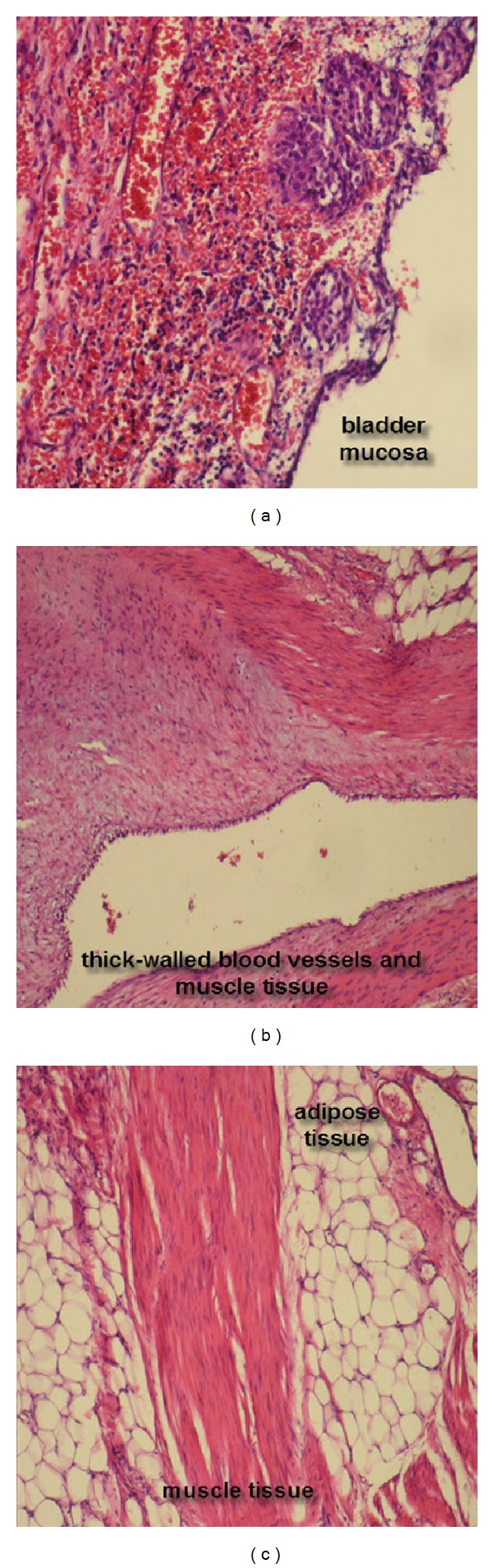
Histology of bladder mass. Tumor is composed of adipose tissue, thick-walled blood vessels, and muscle tissue. H&E ×200.

Morphology is similar in the samples taken from both regions. Wide, normochromatic, and eccentrically located lipoma areas with a clear cytoplasm and nucleus consisting of lipocytes and scattered smooth muscle fibrils and thick wall structures are observed on the sections. In the trichrom staining, muscle fibrils are observed to show activation with dark red. Mitotic activity is not prominent. Pleomorphism and atypia are not observed. In the immunohistochemical examination, positive staining is observed in the epithelial area and vascular endothelia with cytokeratin, while positive staining is observed in the smooth muscle fibrils with actine and negative staining is monitored in connective and fatty tissues with vimentine, whereas negative staining is observed with S100 (Figure 3).

## 5. Discussion

 TS is an inherited neurocutaneous disorder that is characterized by pleomorphic features involving many organ systems, including multiple benign hamartomas of the brain, eyes, heart, lung, liver, kidney, and skin [4]. Angiomyolipoma is classically described as a benign tumor composed of three elements in variable proportion: smooth muscle, adipocytes, and thick-walled blood vessels [5].

We could not find in the literature reported bladder mass lesions in TS. A few number of angiomyolipoma cases originated from the bladder wall have been reported, although these were not related with TS [6].

Most of the patients with TS have one or more skin lesions, as seen in our patient. Other lesions suggesting TS are cerebral and cardiac hamartomas and renal angiomyolipomas, also seen in our patient. 

 There are some reported cases which have isolated bladder angiomyolipoma. One of the angiomyolipomas was established with cystoscopy, which was 1 cm in size and detected with sonography [5]. Similarly, Nicholson et al. presented a case with bilateral renal angiomyolipoma and pelvic mass, causing retroperitoneal bleeding. Pelvic mass was resected through robotic laparoscopic surgery and pathological diagnosis was set for angiomyolipoma [7]. All reported cases did not mentioned about association of angiomyolipomas with TS. This is the first case report that mentioned bladder angiomyolipoma concomitant with TS.

Two types of renal angiomyolipoma are recognized: (1) the sporadic type, usually a unilateral neoplasm typically seen in middle-aged women, (2) the multifocal neoplasm, usually small and bilateral, diagnosed in young men in association with TS [8]. There were bilateral renal angiomyolipomas in our case. However, unlike this reported case in the literature, in our patient angiomyolipoma was of a large size (12 cm) enough to cause retroperitoneal bleeding. 

## 6. Conclusion

We present a rare case of angiomyolipoma of the bladder in the patient with tuberous sclerosis. Diagnosis was confirmed by the histologic and immunohistochemical findings.
